# Supplementary material for: Health of Young Adults Experiencing Social Marginalization and Vulnerability: A Cross-National Longitudinal Study
Source: Int J Environ Res Public Health. 2023 Jan 17;20(3):1711. doi: 10.3390/ijerph20031711 (PMC9914820; doi:10.3390/ijerph20031711)
Supplement: Supplementary file 1 [file ijerph-20-01711-s001.zip › ijerph-2092807-supplementary.pdf]

Supplementary Materials

Table S1. Univariate regression models of age 25 social vulnerability and marginalization associations with general and physical health outcomes at age 28.

	**Poor Perceived Health Status**		**Chronic Illness**		**Serious Injury**		**Disability or Ongoing Medical Problems Resulting from Serious Injury**	
	**b (SE)**	**p**	**B (SE)**	**p**	**B (SE)**	**p**	**B (SE)**	**p**
Social vulnerability and marginalization								
Homelessness	0.20 (0.11)	0.058	0.29 (0.25)	0.231	**0.94** (0.37)	**0.012**	0.12 (0.61)	0.846
ACEs	**0.08** (0.01)	**<0.001**	**0.14** (0.03)	**<0.001**	**0.14** (0.05)	**0.006**	0.11 (0.06)	0.057
LGBT	**0.32** (0.07)	**<0.001**	**0.28** (0.14)	**0.041**	−0.18 (0.30)	0.558	−0.32 (0.39)	0.412
Financial insecurity	**0.17** (0.02)	**<0.001**	**0.19** (0.05)	**<0.001**	**0.25** (0.09)	**0.005**	0.18 (0.13)	0.182
Justice system involvement	0.16 (0.09)	0.090	0.34 (0.19)	0.070	**1.08** (0.31)	**0.001**	**0.91** (0.39)	**0.018**
Demographic factors								
Age	0.10 (0.06)	0.104	0.09 (0.13)	0.465	0.32 (0.23)	0.171	0.39 (0.24)	0.105
Male	−0.08 (0.05)	0.135	−0.18 (0.11)	0.123	0.35 (0.23)	0.128	0.25 (0.28)	0.372
Nonwhite	−0.11 (0.08)	0.194	−0.24 (0.17)	0.163	0.27 (0.30)	0.378	0.04 (0.39)	0.924
Washington State	−0.08 (0.05)	0.118	**−0.29** (0.11)	**0.010**	0.36 (0.23)	0.114	0.51 (0.28)	0.071
Family SES ^	**−0.40** (0.06)	**<0.001**	**−0.32** (0.13)	**0.012**	**−0.70** (0.24)	**0.004**	−0.01 (0.31)	0.989

Note. Age was centered at the mean. Bolded effects are significant at $p < 0.05$. SES: socioeconomic status; b = unstandardized regression coefficient; B = log-odds regression coefficient; SE: standard error. ^ measured in adolescence.

Table S2. Univariate regression models of age 25 social vulnerability and marginalization associations with mental and substance use outcomes at age 28.

	**Self-reported Depression and Anxiety Symptoms**		**Diagnosed Mood Disorder**		**Diagnosed Anxiety Disorder**		**Diagnosed Substance Use Disorder**	
	**b (SE)**	**p**	**B (SE)**	**p**	**B (SE)**	**p**	**B (SE)**	**p**
Social vulnerability and marginalization								
Young adult homelessness	**0.49** (0.13)	**<0.001**	0.34 (0.27)	0.201	**0.60** (0.26)	0.018	**1.78** (0.39)	**<0.001**
ACES	**0.15** (0.01)	**<0.001**	**0.29** (0.03)	**<0.001**	**0.24** (0.03)	**<0.001**	**0.30** (0.05)	**<0.001**
LGBTI	**0.55** (0.07)	**<0.001**	**1.07** (0.14)	**<0.001**	**0.96** (0.14)	**<0.001**	**1.01** (0.32)	**0.002**
Financial insecurity	**0.23** (0.03)	**<0.001**	**0.34** (0.06)	**<0.001**	**0.29** (0.06)	**<0.001**	**0.58** (0.11)	**<0.001**
Justice involvement	**0.28** (0.11)	**0.008**	−0.24 (0.24)	0.305	0.22 (0.21)	0.308	**1.98** (0.38)	**<0.001**
Demographics								
Age	0.04 (0.06)	0.578	−0.10 (0.14)	0.496	**−0.31** (0.14)	**0.025**	0.02 (0.30)	0.951
Male	**−0.24** (0.06)	**<0.001**	**−0.78** (0.14)	**<0.001**	**−1.16** (0.14)	**<0.001**	−0.21 (0.33)	0.528
Nonwhite	−0.11 (0.08)	0.186	**−0.42** (0.20)	**0.039**	−0.32 (0.20)	0.100	0.17 (0.42)	0.691
Washington	−0.08 (0.06)	0.167	−0.07 (0.13)	0.600	0.06 (0.13)	0.633	**1.59** (0.39)	**<0.001**
Family SES	**−0.23** (0.07)	**0.001**	−0.09 (0.15)	0.530	0.08 (0.14)	0.550	−0.27 (0.29)	0.367

Note. Age was centered at the mean. Bolded effects are significant at $p < 0.05$. SES: socioeconomic status; b = unstandardized regression coefficient; B = log-odds regression coefficient; SE: standard error.
